# Nodular Calcification in Saphenous Vein Graft Successfully Treated by Percutaneous Coronary Intervention

**DOI:** 10.1155/2018/5138705

**Published:** 2018-11-08

**Authors:** Ko Yamamoto, Masahiro Natsuaki, Takeshi Serikawa, Masanori Okabe, Yusuke Yamamoto

**Affiliations:** ^1^Division of Cardiology, Cardiovascular and Aortic Center, Saiseikai Fukuoka General Hospital, Fukuoka, Japan; ^2^Department of Cardiovascular Medicine, Saga University, Saga, Japan; ^3^Division of Cardiology, Wajiro Hospital, Fukuoka, Japan

## Abstract

Nodular calcification is sometimes detected in the native coronary artery. However, it is very rare to find in a saphenous vein graft (SVG). We herein report a rare case of stable angina pectoris (AP) due to nodular calcification. A 75-year-old man who had previously undergone coronary artery bypass grafting was admitted to our hospital due to stable AP. On angiography, significant stenosis was detected in the proximal SVG. Based on the findings of coronary angiography and optical coherence tomography, a red thrombus was suspected at the culprit lesion. However, nodular calcification was also suspected, as there were calcifications around the lesion. As intravascular ultrasound showed the protruding calcification, which we judged to be a nodular calcification, the calcified SVG lesion was successfully treated by percutaneous coronary intervention without any complications. Nodular calcification should be considered as a potential cause of AP, even when located in a SVG.

## 1. Introduction

Nodular calcification is sometimes detected in the native coronary artery but is very rarely located in a saphenous vein graft (SVG). We herein report a rare case of stable angina pectoris (AP) due to nodular calcification in a SVG successfully treated by percutaneous coronary intervention (PCI).

## 2. Case Presentation

A 75-year-old man was admitted to our hospital due to stable AP. Coronary artery bypass grafting (CABG) had been performed 15 years earlier. The left internal thoracic artery (LITA) and SVG were anastomosed to the left anterior descending artery (LAD) and right coronary artery, respectively. He also had diabetes, hypertension, and hemodialysis.

**Figure 1:**
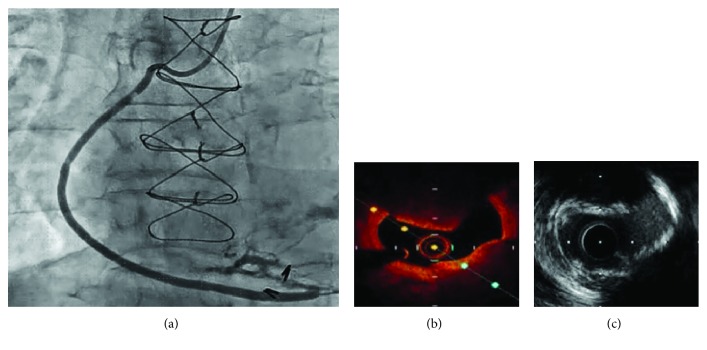
(a) Coronary angiography of saphenous vein graft. (b) OCT finding at the lesion. (c) IVUS finding at the lesion.

On coronary angiography, the right coronary artery and LAD were totally occluded. There was no significant stenosis in the left circumflex. Regarding the bypass graft, the LITA-LAD was patent but the SVG had significant stenosis at the proximal site (Figure 1(a)). Therefore, the SVG lesion was considered to be the culprit lesion for AP.

The SVG stenosis appeared to be a thrombotic lesion on angiography, despite the presence of stable AP. To confirm the lesion characteristics, we performed optical coherence tomography (OCT). The OCT findings also suggested a red thrombus with attenuation (Figure 1(b)). However, nodular calcification was also suspected, as there were calcifications around the lesion. Therefore, intravascular ultrasound (IVUS) was also performed to distinguish the red thrombus from the nodular calcification. As protruding calcification was detected by IVUS (Figure 1(c)), the lesion was judged as not the red thrombus but the nodular calcification. There were no diffuse degenerative plaques at the culprit lesion according to the OCT and IVUS findings.

**Figure 2:**
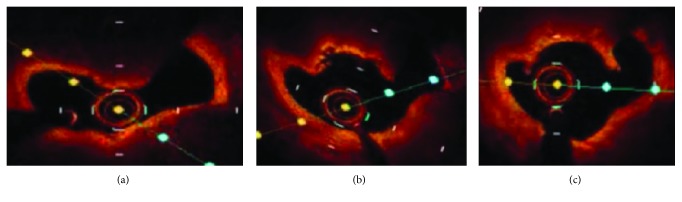
(a) OCT findings before rotablator. (b) OCT findings after ablation with 1.75 mm rotablator burr. (c) OCT findings after ablation with 2.0 mm rotablator burr.

**Figure 3:**
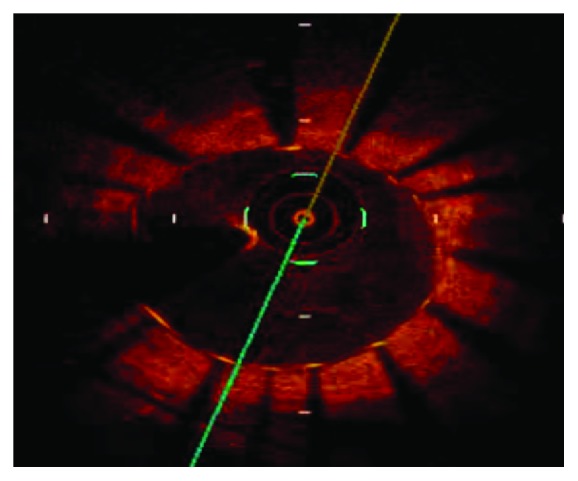
OCT findings after stenting.

**Figure 4:**
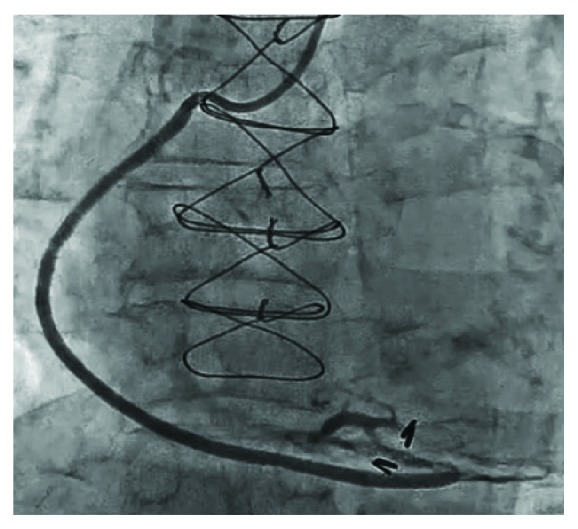
Final coronary angiography.

Rotational atherectomy was considered for the treatment of this focal and protruding calcified lesion, although the use of a rotablator for a diffuse degenerative SVG lesion is basically contraindicated.  Figure 2(a) shows the OCT findings preablation, and Figures 2(b) and 2(c) show the postablation OCT findings using 1.75 and 2 mm rotablator burrs, respectively. After ablation with a rotational atherectomy device, predilation with a scoring balloon (NSE 3.0 × 13 mm, Goodman Co., Ltd.) and stenting (Promus 3.5 × 16 mm, Boston Scientific) were performed. A well-apposed and expanded stent was confirmed based on the poststenting OCT findings (Figure 3). The nodular calcification in the SVG was successfully treated by PCI without any complications (Figure 4).

## 3. Discussion

A SVG is commonly used as a conduit for CABG. However, SVGs are associated with poor long-term patency rates after CABG. SVG disease can be present in approximately 30% of patients 5 years after CABG [1]. Reoperation of CABG is reported to carry an increased risk of periprocedural death and myocardial infarction [2]. Therefore, PCI should be considered for the treatment of SVG lesions in order to avoid reoperation.

However, SVG intervention remains technically challenging and is also associated with increased rates of periprocedural myocardial infarction, in-hospital mortality, restenosis, and occlusion compared with PCI of native coronary arteries mainly because of the friable, degenerated atheromatous, and thrombotic debris that develop when SVGs deteriorate [3, 4]. Foam cell infiltration can be observed within 1 year of SVG implantation, with subsequent necrotic core formation and rupture ensuing after seven years in over one-third of patients [5]. Therefore, SVG lesions may be the culprit for acute coronary syndrome [6]. Calcification is also seen in the SVG as a result of atherosclerosis, being reportedly found in 40% of patients undergoing SVG PCI according to IVUS findings [7]. The present case was not one of acute coronary syndrome but stable AP with calcified lesion detected by IVUS.

Nodular calcification is calcified plaque projecting into the vessel lumen and is sometimes detected in the native coronary artery [8]. However, nodular calcification resembles a red thrombus on coronary angiography and OCT [9]. Therefore, it is important to distinguish the red thrombus from the nodular calcification when we perform PCI. Both nodular calcification and organized thrombus are identified as a protruding mass with signal attenuation on OCT. In the present case, IVUS findings were helpful in distinguishing the red thrombus from the calcification. IVUS depicted a bright protruding mass with marked posterior shadowing, hallmarks suggestive of a heavily calcified plaque. As nodular calcification mimics red thrombus on OCT, we should carefully interpret intravascular images and be aware of the diagnostic accuracy of OCT [10].

Debulking devices should be considered in the treatment of calcified lesions. However, nodular calcification in the SVG is very rare, and PCI for this lesion using rotational atherectomy might be challenging. Thomas et al. reported that 17 calcified lesions in the SVGs were treated in 14 patients using a rotational atherectomy device and that TIMI 3 flow was present in all patients before and at the completion of the intervention [11]. Transient slow-flow phenomenon occurred in 1 of 17 lesions (6%) in association with chest pain and ST segment elevation. One patient had a dissection complicated by transient abrupt closure of the SVG distal anastomosis, which was successfully treated with prolonged balloon inflation. No distal emboli or persistent vessel spasm was seen. Procedural success was achieved in 100% of patients. Therefore, ablation by the rotational atherectomy device may be an option for managing nodular calcification, even in SVG.

In the present case, SVG stenosis resembled a thrombotic lesion on coronary angiography and OCT. However, IVUS showed protruding calcification, suggesting it was nodular calcification. The appropriate use of imaging modalities was helpful for achieving an accurate diagnosis and performing the optimal treatment in this rare case of nodular calcification in a SVG.

## 4. Conclusion

Nodular calcification was detected in a SVG 15 years after CABG. Nodular calcification should be considered as a potential cause of AP, even when located in a SVG.
